# Solar salt lake as natural environmental source for extraction halophilic pigments

**Published:** 2010-06

**Authors:** A Khanafari, D Khavarinejad, A Mashinchian

**Affiliations:** 1 Microbiology Department, North of Tehran Branch, Islamic Azad University; 2 Marine Biology Department, Science and Research Branch, Islamic Azad University

**Keywords:** Halorubrum sodomense, haloarchaea, salt lake, metarhodopsin II, pigment

## Abstract

**Background and Objectives:**

Halophilic bacteria produce a variety of pigments, which function as immune modulators and have prophylactic action against cancers. In this study, colorful halophilic bacteria were isolated from solar salt lake and their pigments was extracted in optimal environmental conditions and compared with the pigments of *Halorubrum sodomense* ATCC 33755.

**Materials and Methods:**

Water samples from the solar salt lake in Imam Khomeini port in southwest of Iran were used as a source for isolation of pigment-producing bacteria. *Halorubrum sodomense* ATCC 33755 was used as control for pigment production. The conditions for optimum growth and pigment production were established for the isolated bacteria. Pigment were analyzed by spectrophotometer, TLC and NMR assay. The 16S rRNA genes were sequenced and results were used to differentiate haloarchaea from halophilic bacterial strains.

**Results:**

Among the isolated strains, YS and OS strains and Halorubrum sodomense were recognized as moderate and extremely halophile with maximum growth in the presence of 15% and 30% NaCl concentrations, respectively. Experiments conducted to find out the optimum conditions for growth and pigment production temperature at 25°C, pH = 7.2 and shaking conditions at 120 rpm for three strains. Without shaking, little growth with no pigment production was observed. Total pigment produced by red, yellow and orange strains was measured at 240, 880 and 560 mg per dry cell weight respectively. Amplification yielded bands of to isolated strains only observed with bacteria primers. This result suggesting the YS and OS strains were not haloarchaea.

**Conclusion:**

The isolated halophilic bacteria produced much higher amounts of pigments than *Halorubrum sodomense.* Photo intermediates including metarhodopsin II (meta II, λ_max_=380 nm) were determined as major pigment in *Halorubrum sodomense*.

## INTRODUCTION

One of the most remarkable biological phenomena in nature is coloration of salt lakes that occur in arid regions throughout the world. The coloration is caused by astronomical numbers of microscopic, unicellular salt-loving bacteria, called Halobacteria living in the water and salt crust. Halophilic bacteria thrive in saline lakes with salt concentrations of 15 to 30 per cent. This is roughly four to nine times the salinity of sea water (3.5 percent). They can even live in saturated salt and survive in salt crystals for years. Whilst the optimum condition for growth is 20 to 30 percent salinity, they cannot survive if the concentration drops much below 12 percent. Very few life forms on earth are known to be adapted to this extreme salinity (1, 2).

Most halophilic bacteria show red color due to the presence of carotenoids. The bacteria produce a red carotenoid pigment which is similar to that found in tomatoes, red peppers, pink flamingos, and in many colorful flowers and autumn leaves. Carotenoid pigments are also the source of Beta-carotene, an important antioxidant and the precursor of vitamin A (3–6). In fact, in some parts of the world, Beta-carotene is extracted from salt ponds containing red salt-living bacteria and algae. In the case of Halobacteria living in salt lake, the red pigment may protect their delicate cells from the intense desert sunlight (7). Rhodopsin- films are superior optical recording materials that combine the advantages of high resolution silver halide films with real-time properties (8).

Isoprenoid derivatives like as carotenoids (C40), bacterioruberins (C50 analogs of carotenoids) and diphytanyl-glycerol, were found in *Halobacterium* spp. Four kinds of retinal-containing chromo-proteins in the cell membrane of *Halobacterium salinarium* were found. Two of them are light-driven ion pumps which convert light energy into chemical energy. Bacteriorhodopsin (bR) transports protons from inside to outside of the cell and halorhodopsin (hR) transports chloride anions from outside to inside (9, 10). These ions pumping generate the transmembrane difference in the electrochemical potential for ions. A cycle of conformational changes of the ion pumps driven by photoexcitation transports ions. The cycle begins with the photoisomerization of retinal from *ali-trans* to 13-cis form (11, 12). This cyclic conformational change, called photocycle, is spectroscopically detected as a series of intermediates with distinct absorption maxima (13, 14).

Halophilic bacteria were originally of interest because of the striking changes they caused in the landscape by imparting various shades of red to natural salterns, spoiled foods and discolored hides. Halophiles produce a variety of red pigments, which impart color to the environment in which they are found. Due to their specific role as immune modulators and prophylactic action against cancers, these protein molecules gain importance in recent research activities. The biotechnological uses of bacterial pigments are only poorly understood (2).

The present study was undertaken to isolation, purification and characterization of colorful haloarchaea or halophilic bacteria strains to find a new source of pigments from solar salt lake in Imam Khomeini port. To maximize the yield of pigment production by the isolated strains and the optimum conditions for the production were also investigated.

## MATERIALS AND METHODS

This research was carried out in the Marine Biology Department, Science and Research branch Laboratory*,* Islamic Azad University, Tehran-Iran. All data reported in this study are from triplicate measurement.

**Bacterial isolates, culture media and growth conditions.** Three colorful strains were used: two halophile strains (YS and OS) were isolated from the solar salt lake in Imam Khomeini port in southwest of Iran and *Halorubrum sodomense* ATCC 33755 (transfer of *Halobacterium sodomense*) was originally obtained from Persian Type Culture Collection (PTCC), Tehran-Iran.

Cultures were grown on Halobacteria medium (5g yeast extract, 5g casamino acids, 1g Na-glutamate, 2g KCl, 3g Na -citrate, 20g MgSO .7H O, 200g NaCl, 36mg FeCl. 4H O, 36mg MnCl. 4 H O, 1000mL distilled water, pH was adjusted to 7.0-7.2) and Nutrient broth containing 100 g/L NaCl, in shaking incubators at 25°C and 120 rpm for 20 days in capped 250-mL Erlenmeyer flasks containing 75mL of each medium. The cell density in the culture was monitored with UV-VIS scanning spectrophotometer, UV2101 pc, Shimadzu by measuring the absorbance at 600 nm (15).

**Salt-tolerance levels.** Three halophile strains were tested to determine the minimal inhibitory concentrations (MICs) and minimal bactericidal concentrations (MBCs) for sodium chloride solutions. The experimental tubes were prepared by supplementing Halobacteria medium (HM, DSMZ medium 372) containing 0-350 g/L NaCl. One milliliter of the test organism suspension (OD_600_=0.8-1, 1×10^6^ CFU/mL) was added to each tube. The tubes were incubated for 20 days at 25°C, 120 rpm and visual turbidity was noted. An aliquot of 0.1 mL from nonturbidal tubes was subcultured to agar for determining MBC (16).

**Bacterial growth curve.** Each of the isolated strains was grown in 50mL Nutrient broth containing 100g/L NaCl in a 250mL Erlenmeyer flask and incubated on a rotary shaker at 120 rpm for 12 days at 25°C. *Halorubrum sodomense* ATCC 33755 was grown in HM broth with 300g/L NaCl. The bacterial growth was measured every 24h in terms of optical density at 600nm with UV-VIS scanning spectrophotometer, UV2101 pc, Shimadzu (15).

**Optimization of culture conditions.** Determination of optimum conditions for growth and pigment production was carried out by inoculating the selected strain in 100mL HM broth in a 500mL Erlenmeyer flask and incubating on a rotary shaker at 120 rpm for 20 days at 25°C. Growth and pigment production were determined as described below. Parameters such as NaCl (concentration was adjust between 0, 10, 20, 30, 40, 50, 60, 70, 80, 90, 100 … 350 mg/L), shaking condition (0, 80, 100, 120 and150 rpm); temperature (15, 20, 25, 30, 37, 40 and 45°C); sources (glucose and sucrose (1%)), NH NO and yeast extract (1%) (inorganic and organic nitrogenous sources respectively) and pH (5, 6, 7.2, 7.5, 8 and 8.5) were tested. The optimum parameter from each experiment was utilized in the subsequent assay until all the parameters were optimized (15).

**Pigment extraction and analysis.** Each of the isolated strains was grown in 50mL Nutrient broth containing 100g/L NaCl, in a 250 mL Erlenmeyer flask. *Halorubrum sodomense* ATCC 33755 was grown in HM broth with 300 g/L NaCl and incubated on a rotary shaker at 120 rpm for 20 days at 25°C. When the culture reached the early stationary phase, according to growth curve, cells were harvested by centrifugation (10000 rpm, 15 min), washed twice with 4M NaCl solution at the centrifuge and finally re- suspended in 4M NaCl solution. The pigments were extracted by acetone, methanol and hexane solvents.

Pigments were analyzed by scanning the absorbance in the wavelength region of 200-800 nm using UV- VIS scanning spectrophotometer (UV 2101 pc, Shimadzu). The total pigment content in solvents extract was estimated by measuring the absorbance at 490nm. The highest pigment-producing strain was selected on the basis of high growth, pigment and degree of cell pigmentation (17).

**Purification of pigment by thin layer chromatography.** Concentrated pigment extract was subjected to TLC using silica gel 60 F MERCK TLC paper. The extract was spotted on TLC plates along with standard carotenoid and eluted with a mobile phase of hexane: methanol (1:1) and ethanol: hexane (7:3) (18).

**IR analysis.** The fraction of TLC paper which had a fluorescence characteristic stronger than others after determined Rf value was scrapped and dissolved in acetone and the peaks were obtained by IR assay (19).

**NMR analysis.** The fluorescence fraction was obtained from the TLC was dissolved in acetone and the NMR spectrum was obtained. The NMR equipment had V: 300 MHz, B 0:7 T (Bruker, UK) (19).

**Molecular analysis.** Total DNAs of YS and OS strains were extracted by the method of Cline *et al* (20) and DNA extraction kit (Sigma, St. Louis, USA). The 16S rRNA gene of the isolate was amplified using universal primers with the following forward and reverse primers for bacteria [5′-AGAGTTTGATCCTGGCTCAG-3′ (8F) and 5′-GACTACCAGGGTATCTAATC-3′ (805R)] and archaeal [5′-ATTCCGGTTGATCCTGCCGG-3′ (6F) and 5′-AGGAGGTGATCCAGCCGCAG-3′ (1540R)]. The amplification was performed by initial denaturation at 95°C for 5 min followed by 10 cycles of 93°C for 1 min, 63°C for 1 min, 71°C for 1.5 min; 20 cycles of 93°C for 1 min, 67°C for 1 min, 71°C for 2 min; and a final extension at 71°C for 5 min.

## RESULTS

Two halophilic strains isolated from solar salt lake in Imam Khomeini port with the highest potency for pigment production were selected. Both of isolated organisms produced amplicons when bacterial specific primers were used in amplification. The results of DNA sequencing confirmed the identity of these strains as bacteria.

Maximum peaks for orange and yellow strains pigments (OS and YS strains) obtained at 208 and 211 nm**,** respectively. The spectra of *Halorubrum sodomense* ATCC 33755 pigments is characterized by maximum peaks at 393 and 359 nm.

Table 1 summarizes the growth and pigment production by halophile strains.

**Table 1:** Growth rate and pigment production by halophile strains in maximum rate.

Strains	Growth (OD_600nm_)	Pigment (OD_490nm_)	Pigment (mg per dry cell weight)
***Halorubrum sodomense*** ATCC 33755	1.392	0.689	240
**strain YS**	1.676	0.980	880
**strain OS**	1.461	0.725	560

**Fig. 1:**
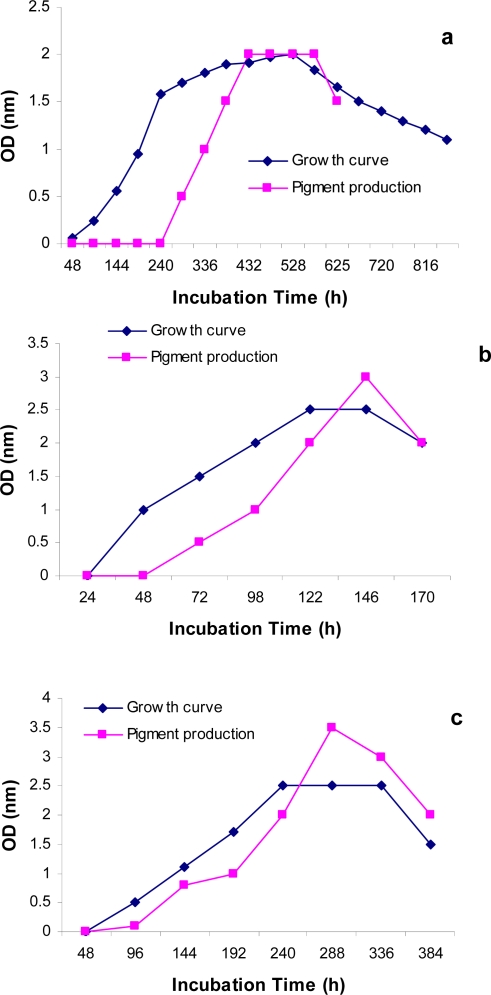
Growth and pigment production; a) *Halorubrum sodomense* ATCC 33755, b) strain YS *(yellow),* c) strain OS *(orange)*: growth (OD_600mm_), pigment (OD_490nm)_.

As shown in Fig. 1-a, the growth curves of *Halorubrum sodomense* ATCC 33755 was characterized by a lag phase about two days. Pigment production by this strain was observed after 10 days of incubation, as the culture became slightly orange in color. Growth and pigment production increased to a maximum after 18-20 days of incubation, followed by a decrease in both growth and pigment production. Fig. 1 (a-c) shows the maximum growth and pigment production after 12 days by yellow and orange strains, respectively.

The growth and pigment production by *Halorubrum sodomense* did not occur when the concentration of NaCl was <15%, but pigment production was in maximum when the concentration was 30%. Conversely, the growth and pigment production by the isolated isolated organism did not occur when the concentration of NaCl exceeded 15%. They could grow in media without salt

**Fig. 2:**
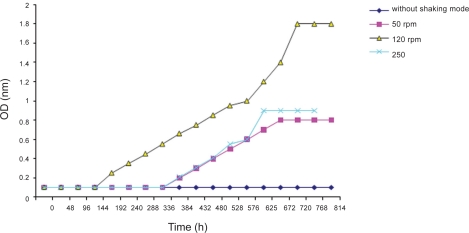
Effect of agitation on the growth of *Halorubrum sodomense* cells in poor agitation, moderate agitation (120 rpm) and vigorous agitation (250 rpm). Cells were grown in Halobacterium medium at 25°C.

When the strains cultivated under shaking conditions at 120 rpm, they exhibited high growth and pigment production. Without shaking, little growth and no pigment production was observed. The effect of agitation on the growth of *Halorubrum sodomense* cells was shown in Fig. 2.

The growth of these strains and pigment production did not occur at pH< 5 and pH >7.5. Maximum growth and production occurred at pH 7.2-7.5.

**Fig. 3:**
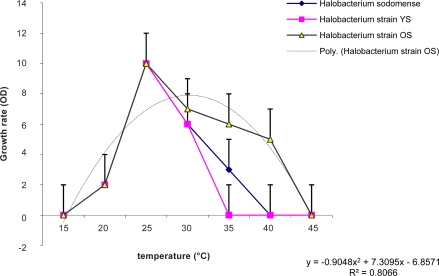
Effect of temperature on growth rate and pigment production of isolated bacteria and control strain, growth (OD 600 nm).

Fig. 3 demonstrates that the growth and pigment production by strains did not occur below 20°C and above 45°C. The trend line displays a general increase with tempreature, with the rate of increase peaking at 25°C. Growth and pigment production by strains decrease when tempreature increase from 25–45°C or decrease from 25°C. In the other hand, the maximum point for growth and pigment production occurred at 25°C.

Optimization of conditions for growth and pigment production showed that when sucrose, as extra carbon source, was added to medium (1%), it gave a higher pigment yield comparing with adding glucose (1%). Sucrose had a significant decreasing effect on bacterial growth curve time from 20 to 3 days and 12 to 2 days for *Halorubrum sodomense* and strains YS and OS, respectively. Addition of NH4NO3 as extra mineral nitrogen source had an amplifying effect on the growth and pigment yield, the same as adding yeast extract.

The alumina column (in TLC method) after development with hexane-methanol (1:1) and ethanol-hexane (7:3), showed only one green fluorescent band when viewed under ultraviolet light (254 and 366 nm). Rf values for red, yellow and orange extracted pigments were 0.434, 0.333 and 0.263 respectively.

Strains, grown in HM and nutrient broth media under optimum conditions, produced 240, 880 and 560mg total pigment per dry cell weight by red, yellow and orange strains, respectively.

IR spectrum result showed that, a characteristic absorbance at 1713.78 cm-1 was assigned to C = O bond and the other peak in the spectrum belong to C-H and C=C in yellow strain. There was no C = O bond observed by *Halorubrum sodomense* and orange strain instead of the two last bacteria C-O-C bond determined at 1110.53 and 1113.93**,** respectively.

Results obtained from NMR analysis showed, a characteristic absorbance was assigned to methyl group in 2.07 ppm area, methyl-allyle groups at 1.21 ppm area and methyl-vinyl groups at 1.59 ppm area. Also O-H group and vinyl group were observed at 2.07 and 5.39 ppm area.

All of yielded amplification bands only with bacteria primers.

## DISCUSSION

In this study both extremely and moderate halophilic strains were isolated from solar salt lake in southwest of Iran, showed good potential for producing pigments. The spectra of *Halorubrum sodomense* ATCC 33755 pigments is characterized by maximum peaks at 393 and 359 nm. A series of bounded and unbounded retinal-containing chromo-proteins in the cells of *Halobacterium* spp. were reported (10). The most famous of unbounded retinal pigments which observed in genus of halobactrium include bathorhodopsin (batho, λ_max_=543 nm) and lumirhodopsin (lumi, λ_max_= 497nm) (21), metarhodopsin I (meta I,_max_=480 nm) and metarhodopsin II (meta II, λ=380 nm) (8, 22) (23). The spectera of *Halorubrum sodomense* ATCC 33755 pigments is similar to absorption peak of unbound retinal pigment spectrum such as metarhodopsin II. According to Fisher and Weiss 1974 and Stoeckenius *et al*. (1979), the absorption peak of unbound retinal is between 350 and 380 nm, depending on the solvent and retinal configuration. The remaining pigments were not identified (24, 25).

It is very interesting that YS strain isolated in this study produced more pigment (880 mg per dry cell weight) than *Phafia rhodozyma* (410 mg/L)and *Agrobacterium auranticum* (460 mg/L) (26).

The experiment conducted to prove the essentiality of oxygen for growth and pigment production showed that shaking grown condition was more preferable to the test organism than the static condition. Many halophilic and aerobic marine strains were reported to produce pigments (27, 28).

Maximum growth and pigment production occurred at pH 7.2-7.5. It seems that acidic or alkaline pH may have an inhibitory effect on bacterial growth and production.

The trend line displays a general increase with tempreature, with the rate of increase peaking at 25°C. Growth and pigment production by strains decrease when tempreature increase from 25–45°C or decrease from 25°C. In the other hand, the maximum point for growth and pigment production occurred at 25°C. So these strains don't belong to psychrophilic or thermophilic microorganisms.

Further experiment on the response of the test organism against different carbon sources, showed interesting results. The growth of the test organism was drastically stimulated by sucrose. The growth curve of *Halorubrum sodomense* was completed at 3 days instead of 20 days. The growth rate of Halophilic bacteria is much slower than most other bacteria. So it is the most problem to produce metabolite by these bacteria. The addition of compounds solution increased both growth and pigment production of the strain, and reduced generation time. However, one exception is provided by the halophilic organisms, which must accumulate high cellular levels of inorganic ions to cope with extremely high salt concentrations in their environment. The mechanisms of cellular adaptation preventing water loss under hyperosmotic conditions (osmoregulation) have been extensively studied in bacteria, fungi, algae, plants, and animals (29–32). A consensus strategy of osmoregulation leads to the intracellular accumulation of inorganic or organic low-molecular-weight compounds such as carbohydrates (sugars and polyols such as trehalose and glycerol), amino acids (glutamate, proline, pipecolate and ectoine), betaines and their analogues known as compatible solutes (33, 34).

Most of halophilic strains are fastidious and need a complex media. Therefore, their growth and pigment production in simple media such as Nutrient Broth could be attractive. Kannan, 2004 reported good growth for *Planococcus halophilus* inn Nutrient Broth with kerosene and diesel (1%) (35).

The photointermediate pigment (metarhodopsin II) is specifically seen in characteristic bands at 1745, 1713, and 1644 cm-1. These major bands are the critical different in reflecting changes in protonation states, hydrogen bonding, the secondary structure of the protein, and also the chromophore fingerprint between metarhodopsin I and II (36, 37).

## References

[1] Grant WD, Gemmel RT, McGenity TJ (1998). Extremophiles: Microbial life in extreme environments.

[2] Singleton P, Sainsbury D (2001). Dictionary of microbiology and molecular biology.

[3] Tomita Y (1983). Immunological role of vitamin A and its related substances in prevention of cancer. Nutr Cancer.

[4] Tee ES (1992). Carotenoids and retinoid in human nutrition. Crit Rev in Food Sci & Nutr.

[5] Palozza P, Luberto C, Ricci P, Sgarlata E (1996). Effect of p-carotene and canthaxanthin on tert-butyl hydroperoxideinduced lipid peroxidation in murine normal and tumor thymocytes. Arch Biochem Biophys.

[6] Delgado-Vargus F, Jimenez AR, Peredes-Lopez O (2000). Natural pigments: carotenoids, anthocyanins and betalains: characteristics biosynthesis, preparation and stability. CRC Crit Rev Food Sci Nutr.

[7] Shun Ichi S, Hiroyuki S, Hiroaki T (1996). Voltage-dependent absorbance change of carotenoids in halophilic Archaebacteria. Biochimica ET Biophysica Acta.

[8] Menon ST, Han M, Sakmar TP (2001). Rhodopsin: structural basis of molecular physiology. Physiol Rev.

[9] Schobert B, Lanyi JK (1982). Halorhodopsin is a light-driven chloride pump. J Biol Chem.

[10] Spudich JL, Yang CS, Jung KH, Spudich EN (2000). Retinylidene proteins: structures and functions from archaea to humans. Annu Rev Cell Dev Biol.

[11] Béjà O, Aravind L, Koonin EV, Suzuki MT, Hadd A, Nguyen LP (2000). Bacterial rhodopsin: evidence for a new type of phototrophy in the sea. Science.

[12] Béjà O, Spudich EN, Spudich JL, Leclerc M, DeLong EF (2001). Proteorhodopsin phototrophy in the ocean. Nature.

[13] Stoeckenius W, Bogomolni RA (1982). Bacteriorhodopsin and Related Pigments of Halobacteria. Ann Rev Biochem.

[14] Bieszke JA, Spudich EN, Scott KL, Borkovich KA, Spudich JL (1999). A eukaryotic protein, NOP-1, binds retinal to form an archaeal rhodopsin-like photochemically reactive pigment. Biochemistry.

[15] Balint Z, Lakatos M, Ganea C, Lanyi JK, Varo G (2004). Nitrate transporting photochemical reaction cycle of the pharaonis halorhodopsin. Biophysical Journal.

[16] Forbes BA (1998). Enterobacteriaceae. Baily and Scott's Diagnostic Microbiology. Baltimor, Mosby.

[17] Calo P, de Miguel T, Sieiro C, Velazquez JB, Villa TG (1995). Ketocarotenoids in halobacteria: 3-hydroxy echinenone and trans astaxanthin. J Appl Bacterial.

[18] Lorenz Todd R (1998). Thin-Layer Chromatography (TLC) system for Natu Rose carotenoids. Natu Rose Technical Bulletin.

[19] Rao RN, Alvi SN, Rao BN (2005). Preparative isolation and characterization of some minor impurities of astaxanthin by high-performance liquid chromatography. J Chromatogr A.

[20] Cline SW, Schalkwyk LC, Doolittle WF (1989). Transformation of the archaebacterium Halobacterium volcanii with genomic DNA. J Bacteriol.

[21] Lewis JW, Van Kuijk FJ, Carruthers JA, Kliger DS (1997). Metarhodopsin III formation and decay kinetics: comparison of bovine and human rhodopsin. Vision Res.

[22] Jonathan JR, Thorsten M, Reiner V, Claudio V, Gebhard FX (2004). Electron crystallography reveals the structure of metarhodopsin I. EMBO J.

[23] Tomioka H, Takahashi T, Kamo N, Kobatake Y (1986). Action spectrum of the photoattractant response of Halobacterium halobium in early logarithmic growth phase and the role of sensory rhodopsin. Biochim Biophys Acta.

[24] Fisher MM, Weiss K (1974). Laser photolysis of retinal and its protonated and unprotonated n-butylamine Schiff base. Photochem Photobiol.

[25] Stoeckenius W, Lozier RH, Bogomolni RA (1979). Bacteriorhodopsin and the purple membrane of halobacteria. Biochim Biophys Acta.

[26] Nelis HJ, Leenheer AP (1991). A review: microbial sources of carotenoid pigments used in foods and feeds. J Appl Bacteriol.

[27] Asker D, Ohta Y (1999). Production of canthaxanthin by extremely halophilic bacteria. J Biosci Bioeng.

[28] Yokoyama A, Miki W (1995). Composition and presumed biosynthetic pathway of carotenoids in the astaxanthin- producing bacterium Agrobacterium aurantiacum. FEMS Microbial Lett.

[29] Garcia-Perez A, Burg MB (1991). Renal medullary organic osmolytes. Physiol Rev.

[30] Imhoff JF (1986). Osmoregulation and compatible solutes in eubacteria. FEMS Microbiol Rev.

[31] Wyn Jones RG, Gorham J (1984). Phytochemical aspects of osmotic adaptation. Recent Adv Phytochem.

[32] Yancey PH, Clark ME, Hand SC, Bowlus RD, Somero GN (1982). Living with water stress: evolution of osmolyte systems. Science.

[33] Rolland JP, Bron P, Thomas D (1997). MACS: Automatic counting of objects based on shape recognition. Comput Appl Biosci.

[34] Kunte HJ (2006). Osmoregulation in Bacteria: Compatible solute accumulation and osmosensing. Eniviron Chem.

[35] Kannan V (2004). Microbial system for environmental management. Botany.

[36] Isele J, Sakmar TP, Siebert F (2000). Rhodopsin activation affects the environment of specific neighboring phospholipids: an FTIR spectroscopic study. Biophys J.

[37] Ritter E, Zimmermann K, Heck M, Hofmann KP, Bartl FJ (2004). Transition of Rhodopsin into the active metarhodopsin II state opens a new light-induced pathway linked to Schiff base isomerization. J Biol Chem.

